# Relationship of sagittal spinal alignment with low back pain and physical performance in the general population

**DOI:** 10.1038/s41598-021-00116-w

**Published:** 2021-10-18

**Authors:** Kazuhiro Hira, Keiji Nagata, Hiroshi Hashizume, Yoshiki Asai, Hiroyuki Oka, Shunji Tsutsui, Masanari Takami, Hiroshi Iwasaki, Shigeyuki Muraki, Toru Akune, Toshiko Iidaka, Hiroshi Kawaguchi, Kozo Nakamura, Munehito Yoshida, Sakae Tanaka, Noriko Yoshimura, Hiroshi Yamada

**Affiliations:** 1 Department of Orthopaedic Surgery, Wakayama Medical University, 811-Kimiidera, Wakayama City, Wakayama 641-8510 Japan; 2 Department of Medical Research and Management for Musculoskeletal Pain, 22nd Century Medical and Research Center, Faculty of Medicine, The University of Tokyo, Bunkyo-ku, Tokyo Japan; 3 Department of Preventive Medicine for Locomotive Organ Disorders, 22nd Century Medical and Research Center, Faculty of Medicine, The University of Tokyo, Bunkyo-ku, Tokyo Japan; 4 Rehabilitation Services Bureau, National Rehabilitation Center for Persons With Disabilities, Tokorozawa City, Saitama Japan; 5 Tokyo Neurological Center, Minato-ku, Tokyo Japan; 6 Towa Hospital, Adachi-ku, Tokyo Japan; 7 Department of Orthopaedic Surgery, Sumiya Orthopaedic Hospital, Wakayama City, Wakayama Japan; 8 Department of Orthopaedic Surgery, Faculty of Medicine, The University of Tokyo, Bunkyo-ku, Tokyo Japan

**Keywords:** Neurological disorders, Epidemiology

## Abstract

Studies have suggested a relationship between sagittal spinal malalignment and low back pain (LBP). The current study investigated the relationship of spinal alignment with LBP and physical performance in 1491 individuals who attended the second follow-up visit of the Wakayama Spine Study. The sagittal vertical axis at C7 (C7 SVA) was measured by a spine surgeon. The occurrence of LBP within one month, pain intensity, Oswestry Disability Index (ODI), and physical performance (grip strength, 6-m walking time, chair stand test, one-leg standing test) were also evaluated. LBP in the previous month was determined using ODI, and indicators of physical performance were measured. The mean C7 SVA was 11.0 ± 42.7 mm and was significantly greater in older participants (*p* < 0.001). LBP was more prevalent in participants with a greater C7 SVA (< 40 mm, 35.7%; 40–95 mm, 47.3%; ≥ 95 mm, 59.4%; *p* < 0.001) and those with a higher ODI score (10.0%, 17.5%, and 29.4%, respectively; *p* < 0.001). Physical performance significantly decreased in participants with a greater C7 SVA (*p* < 0.001). Multiple linear regression analysis revealed that LBP and physical performance were significantly associated with C7 SVA (*p* < 0.001). Thus, sagittal spinal malalignment may lead to LBP and decreased physical performance.

## Introduction

Sagittal spinopelvic malalignment is one of the most prevalent disorders of the aging spine.

The sagittal curvature of the spine and pelvis balance each other to maintain a stable posture and horizontal gaze. Glassman et al. reported that positive sagittal balance was significantly related to clinical symptoms and health-related quality of life in patients with adult spinal deformity^1^. When the sagittal alignment is abnormal, more energy is required to maintain balance without external support^2^. Considering the progressive nature of spinal deformity and the ongoing escalation in numbers of elderly people in most developed countries, there is an urgent need for prevention strategies. Nonetheless, the impact of spinal deformity on physical performance, which is the basic information needed for the prevention of spinal deformity, has not been well characterized. The impact of spinal deformity on physical performance cannot be estimated by hospital surveys because many people already have severely impaired functional status by the time they visit a hospital. Therefore, a population-based study is needed to clarify the relationship between sagittal spinal alignment and physical performance. Whole-spine radiographs are important in diagnosing spinal deformity, but few population-based studies of spinal deformity using whole-spine radiography have been performed. Decreased physical performance is considered a symptom of spinal deformity and can lead to decreased quality of life, especially in elderly patients with kyphotic deformity. Although spinal deformity is commonly seen in asymptomatic elderly participants, it is unclear whether or not physical performance deteriorates in patients with adult spinal deformity. We hypothesized that decreased physical performance would be strongly associated with kyphotic deformity. Therefore, the objective of this study was to examine the association between sagittal spinal alignment parameters and physical performance.

## Results

The baseline characteristics of the 1461 study participants, including anthropometric measurements and visual analog scale (VAS) scores for low back pain/neck pain, are shown in Table 1. There were no sex-related significant differences in age, the Oswestry Disability Index (ODI), or the VAS scores for low back pain and neck pain. Height, weight, body mass index (BMI), and grip strength were significantly greater in men than those in women. Table 2 compares the mean values for sagittal spinal alignment in each age strata in this cohort. The mean sagittal vertical axis at C7 (C7 SVA) was 11.0 ± 42.7 mm, and older participants had a larger C7 SVA (*p* < 0.001). The association between sagittal spinal alignment on radiographs and pain/physical performance is shown in Table 3. Univariate analysis indicated that low back pain was more prevalent in participants with a larger C7 SVA (small, 35.7%; intermediate, 47.3%; large, 59.4%; *p* < 0.001), and these participants also had a higher ODI score (small, 10.0%; intermediate, 17.5%; large, 29.4%; *p* < 0.001). Indicators of physical performance decreased significantly in participants with a larger C7 SVA (*p* < 0.001). Finally, after adjusting for sex, age, and BMI, multiple linear regression analysis was performed with individual measures of physical performance as response variables. The association between sagittal spinal alignment and physical performance is shown in Table 4. These measures of physical performance were significantly associated with C7 SVA (*p* < 0.001).

**Table 1:** Characteristics of male and female participants.

	Male	Female
N	466	995
Age (years)	66.3 ± 13.8	65.3 ± 12.5
Height (cm)	164.7 ± 7.3***	151.8 ± 6.7
Weight (kg)	64.2 ± 11.4***	52.4 ± 8.7
Body mass index	23.6 ± 3.4**	22.7 ± 3.5
Grip strength (kg)	39.1 ± 8.8***	24.5 ± 5.6
ODI (%)	10.9 ± 11.9	12.6 ± 13.6
Presence of low back pain (%)	38.8	37.2
Low back pain (VAS)	13.4 ± 21.6	15.2 ± 13.4
Neck pain (VAS)	4.4 ± 13.3	4.6 ± 14.9

The data are shown as the mean ± standard deviation.

*p < 0.05, **p < 0.01, ***p < 0.001: Significantly different from the values for female (Student’s *t*-test).

ODI, Oswestry Disability Index; VAS, visual analog scale.

**Table 2:** Comparison of mean sagittal spinal alignment values among different age groups in the cohort.

	< 50 (n = 170)	50–59 (n = 256)	60–69 (n = 418)	70–79 (n = 407)	≥ 80 (n = 210)	Overall (n = 1461)
*Overall*						
Th5-12 angle (°)	21.6 ± 7.7	23.5 ± 9.4	25.8 ± 10.2	30.1 ± 11.7	33.8 ± 14.2	27.2 ± 10.9
Lumbar lordosis (°)	− 50.7 ± 10.1	− 47.8 ± 10.3	− 46.4 ± 12.5	− 44.2 ± 14.0	− 38.9 ± 17.8	− 45.4 ± 13.2
Pelvic tilt (°)	13.5 ± 6.9	15.3 ± 7.6	17.3 ± 8.1	20.9 ± 10.1	22.9 ± 10.0	18.3 ± 8.8
Sacral slope (°)	35.8 ± 7.8	33.8 ± 7.6	32.6 ± 8.8	30.3 ± 9.1	27.2 ± 10.7	31.8 ± 8.9
C7 SVA (mm)	− 16.2 ± 24.3	− 4.4 ± 27.7	3.7 ± 33.5	21.1 ± 42.2	46.4 ± 56.6	10.9 ± 38.5
*Men*	N = 56	N = 75	N = 124	N = 123	N = 88	N = 466
Th5-12 angle (°)	21.5 ± 7.2	23.0 ± 9.0	25.3 ± 9.4	28.9 ± 10.8	30.1 ± 10.0	26.3 ± 9.6
Lumbar lordosis (°)	− 47.9 ± 9.1	− 45.2 ± 9.5	− 45.2 ± 11.8	− 45.7 ± 13.4	− 39.3 ± 15.9	− 44.5 ± 12.5
Pelvic tilt (°)	11.5 ± 6.9	14.4 ± 6.6	15.5 ± 6.8	16.0 ± 7.5	19.7 ± 8.4	15.8 ± 7.2
Sacral slope (°)	34.5 ± 6.9	33.3 ± 7.7	32.9 ± 8.8	31.5 ± 7.9	28.3 ± 10.2	31.9 ± 8.5
C7 SVA (mm)	− 10.9 ± 22.9	4.7 ± 30.7	8.2 ± 35.0	13.9 ± 39.2	39.1 ± 54.3	12.7 ± 38.8
*Female*	N = 114	N = 181	N = 294	N = 284	N = 122	N = 995
Th5-12 angle (°)	21.7 ± 8.0	23.7 ± 9.5	26.0 ± 10.6	30.6 ± 12.0	36.4 ± 16.2	27.7 ± 11.4
Lumbar lordosis (°)	− 52.0 ± 10.3	− 48.9 ± 10.4	− 46.9 ± 12.8	− 43.6 ± 14.2	− 38.6 ± 19.1	− 45.9 ± 13.5
Pelvic tilt (°)	14.5 ± 6.7	15.8 ± 8.0	18.1 ± 8.5	23.0 ± 10.3	25.2 ± 10.4	19.5 ± 9.0
Sacral slope (°)	36.5 ± 8.2	34.0 ± 7.6	32.5 ± 8.9	29.7 ± 9.6	26.4 ± 11.1	31.7 ± 9.1
C7-SVA (mm)	− 18.8 ± 24.6	− 8.2 ± 25.6	1.8 ± 32.7	24.2 ± 43.1	51.6 ± 57.9	10.1 ± 38.0

The data are shown as the mean ± standard deviation. Differences in the values of the indices were tested for significance using analysis of variance for comparisons among multiple groups.

C7 SVA, sagittal vertical axis at C7.

**Table 3:** Association between sagittal spinal alignment and pain/physical performance on radiographs.

	Small (< 40 mm) (n = 1192)	Intermediate (40 ≤ SVA < 95 mm) (n = 205)	Large (≥ 95 mm) (n = 64)	*p* value
Age (years)	63.3 ± 12.7	74.3 ± 8.9	79.2 ± 7.0	< 0.001
Sex (male/female)	387/805	60/145	19/45	
BMI	22.9 ± 3.5	23.3 ± 3.4	23.7 ± 3.6	0.14
Neck pain VAS (mm)	4.5 ± 14.2	4.4 ± 16.0	4.1 ± 11.0	0.97
Presence of low back pain (%)	35.7	47.3	59.4	< 0.001
Low back pain VAS (mm)	13.0 ± 21.5	19.3 ± 26.0	29.6 ± 29.4	< 0.001
ODI (%)	10.0 ± 11.3	17.5 ± 15.0	29.4 ± 17.4	< 0.001
*Physical performance*				
Grip strength (kg)	30.1 ± 9.5	25.6 ± 9.0	21.9 ± 6.8	< 0.001
Chair-stand time (s)	8.1 ± 2.7	10.1 ± 4.3	12.0 ± 4.2	< 0.001
6-m walking time (m/s)	1.1 ± 0.3	1.0 ± 0.3	0.7 ± 0.3	< 0.001
One leg standing test (s)	44.8 ± 20.6	24.5 ± 21.1	9.8 ± 11.3	< 0.001

The data are shown as the mean ± standard deviation.

One-way analysis of variance was used to assess differences among different distances of the C7 SVA.

BMI, body mass index; ODI, Oswestry Disability Index; VAS, visual analog scale.

**Table 4:** Association of physical performance (grip strength, chair-stand time, 6-m walking time, and one leg standing test) with C7 SVA.

	Standardized-β	VIF	*p *value
**Model 1: Grip strength**			
Age (years)	− 0.40	1.23	< 0.0001*
Sex (male)	0.72	1.01	< 0.0001*
Body mass index (kg/m^2^)	0.09	1.03	< 0.0001*
C7 SVA (mm)	− 0.07	1.23	< 0.0001*
**Model 2: Chair stand time**			
Age (years)	0.37	1.22	< 0.0001*
Sex (male)	− 0.01	1.02	0.74
Body mass index (kg/m^2^)	0.06	1.03	0.015*
C7 SVA (mm)	0.17	1.23	< 0.0001*
**Model 3: 6-m walking time**			
Age (years)	− 0.39	1.23	< 0.0001*
Sex (male)	− 0.01	1.01	0.65
Body mass index (kg/m^2^)	− 0.05	1.03	0.018*
C7 SVA (mm)	− 0.21	1.23	< 0.0001*
**Model 4: One-leg standing test**			
Age (years)	− 0.58	1.22	< 0.0001*
Sex (male)	0.05	1.02	0.0082*
Body mass index (kg/m^2^)	− 0.13	1.03	< 0.0001*
C7 SVA (mm)	− 0.18	1.22	< 0.0001*

Beta values are shown using multiple regression analysis after adjustment for age, sex, and body mass index.

Multiple regression analysis, with measurements of each physical performance (grip strength, 6-m walking time, chair stand time, or one-leg standing test) as objective variables (from Model 1 to Model 4), was performed to estimate the association of physical performance with C7 SVA after adjustment for age, sex, and body mass index. CI, confidence interval; VIF, variance inflation factor; C7 SVA, sagittal vertical axis at C7.

## Discussion

To our knowledge, the Wakayama Spine Study is the first population-based study to use whole-spine radiographs to clarify the age-related difference in sagittal spine parameters and the association with low back pain and physical performance measures in a large cohort. The mean C7 SVA was 11.0 ± 42.7 mm, and older participants had a larger C7 SVA. Univariate analysis indicated that low back pain was more prevalent in participants with a larger C7 SVA, who also had a higher ODI score. Physical performance measures, including grip strength, chair stand time, 6-m walking time, and the one-leg standing test, were significantly associated with C7 SVA.

In this study, thoracic kyphosis (TK), pelvic tilt, and C7 SVA increased with age, whereas LL and the sacral slope decreased. Notably, we found a marked change in C7 SVA in both men and women in their 70 s and 80 s. A Chinese cohort study reported a similar tendency in C7 SVA change^3^. This study showed that global sagittal parameters and T1-pelvic angle and SVA gradually increased with aging, with a sudden increase in knee flexion angle and ankle dorsiflexion angle after 50 years of age, signifying the recruitment of additional physiological compensatory mechanisms in the lower limb in maintaining the sagittal global balance. Thus, lower-limb compensatory mechanisms may also influence increased C7SVA. Gelb et al. investigated 100 asymptomatic middle-aged and older volunteers and found a correlation between C7 SVA, LL, and age^4^. The association between spinopelvic parameters and age may explain the physiological aging of the spine. Miyatani et al. found a greater decrease in muscle thickness in the trunk and anterior thigh than at other sites^5^. We speculated that this greater decrease in trunk muscle thickness might be attributed to age-related changes in spinal alignment, causing altered patterns of loading to and/or activation of individual muscles in daily life.

The impact of sex on spinopelvic parameters remains controversial. In this study, the rates of increase in TK and C7 SVA were greater in women than those in men. Previous studies have suggested that worsening of the sagittal alignment originates in the pelvis in women and the cervical spine in men among volunteers older than 50 years^6^. Regarding pelvic parameters, Vialle et al. reported significant differences in LL and PI between male and female participants^7^. Conversely, other researchers found no significant sex-related difference in any spinopelvic parameter^8–10^. Although there were statistically significant differences in TK, LL, pelvic tilt, and C7 SVA between men and women in the present study, the sex-related difference in the mean value for each parameter was quite small. Moreover, the individual variations were much larger than the sex-related differences. When considering clinically important differences, further studies should be performed to corroborate this finding.

In this study, we demonstrated an association of sagittal malalignment with low back pain in a large cohort. Dubousset described the theory known as the ‘‘cone of economy’’^2^. Within the center of the cone, the individual may remain in an ergonomically favorable erect position. Our data showed that low back pain was more prevalent in participants with a larger C7 SVA (≥ 95 mm). Larger deviations in the anterior–posterior or lateral plane and the resulting progression of imbalance, which shows the failure of the compensation system, require greater energy expenditure to maintain a standing position. We speculate that the failure of various compensation to maintain sagittal balance would result in low back pain. Our epidemiological findings support this theory.

We also demonstrated an association between C7 SVA and decreased physical performance, including grip strength. Several studies have demonstrated an inverse correlation between spinal extensor muscle strength and hyperkyphosis^11,12^. In the lumbar spine, fatty degeneration and volume loss in the paraspinal muscles have been demonstrated in association with degenerative kyphosis^13^. Katzman et al. reported that weak grip and ankle strength are associated with age-related hyperkyphosis, suggesting that general deconditioning is an important contributing factor in the geriatric population^14^. Imagama et al. indicated that the back muscles and physical abilities influence the maintenance of spinal sagittal alignment and may account for the relationship of decreased muscle strength and gait speed with the risk of falling^15^. However, since theirs was an observational, cross-sectional study, the causal relationship between sagittal spinopelvic parameters and physical performance is still unclear and needs to be investigated in a longitudinal study.

This study has several limitations. First, although more than 1000 participants were included, the sample may not be representative of the general population because the participants were recruited from only two areas of Japan. However, when anthropometric measurements were compared between the participants in this study and the general Japanese population, there were no significant differences in the mean BMI of men (23.71 ± 3.41 vs. 23.95 ± 2.64; *p* = 0.33) as well as female (23.06 ± 3.42 vs. 23.50 ± 3.69; *p* = 0.07). Second, evaluation of the alignment of the cervical spine and/or lower extremities should be included because they also show age-related changes and affect spinopelvic alignment. Further study is needed to identify the causal relationship between the overall alignment of the trunk and physical performance.

In conclusion, to our knowledge, this population-based study is the first to use whole-spine radiographs to investigate age-related differences in sagittal spine parameters and their association with low back pain and physical performance measures in a large cohort. A larger C7 SVA was more prevalent in participants with low back pain and decreased physical performance.

## Methods

### Compliance with ethical standards

This study was conducted in accordance with the Declaration of Helsinki, and the study design was approved by the Ethics Committee of the Wakayama Medical University. All volunteers provided written informed consent for participation.

### Participants

With the approval of our institutional review board, the present study, entitled the Wakayama Spine Study, was performed in a sub-cohort of patients who presented for a second follow-up visit in the Research on Osteoarthritis/Osteoporosis Against Disability (ROAD) study, which was initiated in 2011 as a nationwide prospective study of bone and joint diseases in population-based cohorts and was completed in 2012^16^. In addition to the participants enrolled at the outset of the study, inhabitants of the mountainous and coastal areas in the Wakayama prefecture who were willing to participate in the ROAD survey were also included in the second follow-up visit. A total of 1575 individuals (513 male, 1062 female) presented for this visit. One hundred and fourteen individuals who could not maintain a standing position while undergoing total lateral whole-spine radiography or had other disqualifiers were excluded. Finally, lateral whole-spine radiographs were obtained for 1461 participants (466 male, 995 female).

### Evaluation of low back pain

Low back pain was determined by asking the following question: “Did you experience low back pain on most days (and continuously on at least one day) in the past month, in addition to the current pain?” Participants who answered “yes” were considered to have low back pain. The ODI was used to evaluate problems in daily living due to low back pain. The severity of low back pain in the previous week was evaluated using the VAS score.

### Evaluation of physical performance

Medical data on low back pain and physical performance were collected by an experienced orthopedic surgeon. The following tests were conducted to evaluate physical performance: grip strength, 6-m walking time, step length, chair stand time, and one-leg standing time. Grip strength was measured for each hand using a handgrip dynamometer (Toei Light Co., Ltd, Saitama, Japan). To measure walking speed, the time taken to walk 6 m at a usual pace in a hallway was recorded. Similarly, the 6-m walking time at maximal pace was measured. The time taken for five consecutive chair rises without the use of hands was also recorded. One-leg standing time was measured for each leg using a stopwatch (upper limit, 60 s), and the mean value for both legs was recorded^17^.

### Radiographic evaluation

**Figure 1:**
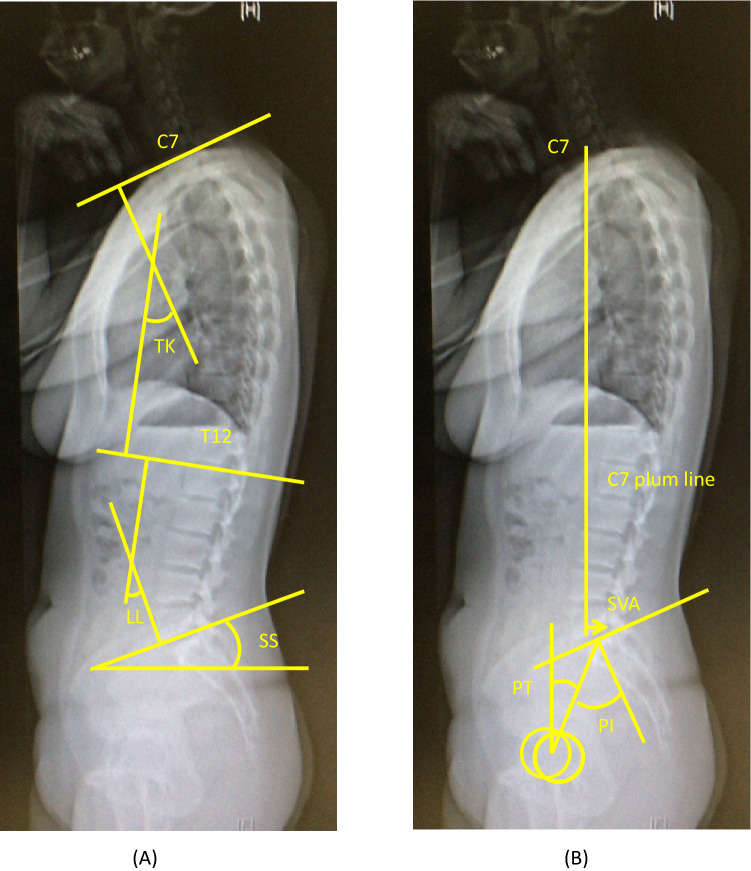
Whole spine radiographic measurements. (**a**) Measurement of thoracic kyphosis (TK), lumbar lordosis (LL) and sacral slope (SS). (**b**) Measurement of diameter of sagittal vertical axis at C7(C7-SVA), pelvic tilt (PT), and pelvic incidence (PI). Pelvic tilt (PT) is defined by the angle between the vertical and the line through the midpoint of the sacral plate to the femoral heads axis (retroversion is then measured as a pelvic tilt increase, anteversion as a pelvic tilt decreases). Sacral slope (SS) is defined as the angle between the horizontal and the sacral plate. Pelvic incidence (PI) is defined as the angle between the perpendicular to the sacral plate at its midpoint and the line connecting this point to the femoral heads axis.

Standing lateral radiographs of the whole spine and pelvis were obtained for each participant using a 40-in. film. Each radiograph was aligned such that the edge of the film was used as a reference for vertical alignment. The participant was instructed to stand in a comfortable position with the hips and knees fully extended. The arms were flexed with the hands resting on the supports at the level of the shoulders. Measured radiographic parameters included the following: TK (the Cobb angle from the upper endplate of T5 to the lower endplate of T12), LL (the Cobb angle from the upper endplate of L1 to the lower endplate of S1), pelvic tilt (the angle between the line connecting the midpoint of the sacral plate to the axis of the femoral heads and vertical axis), pelvic incidence (the angle between the line perpendicular to the sacral plate at its midpoint and the line connecting this point to the axis of the femoral heads), and SVA (the horizontal distance from the C7 plumb line originating at the middle of the C7 vertebral body to the posterior superior endplate of S1) (Fig. 1).

### Statistical analysis

All continuous values are expressed as the mean ± standard deviation. Student’s *t*-test was used to analyze differences in spinal and pelvic parameters between men and women. Participants were divided based on their age into five groups: (1) < 50 years, (2) 50–59 years, (3) 60–69 years, (4) 70–79 years, and (5) ≥ 80 years. Differences in the values of indices were tested for significance using analysis of variance for comparisons among multiple groups. Radiographic parameters were compared among different age groups. The C7 SVA was divided into three categories based on Schwab’s classification^18^ as small (< 40 mm), intermediate (40 ≤ SVA < 95 mm), or large (≥ 95 mm). One-way analysis of variance was used to assess differences among different distances of the C7 SVA. Finally, multiple regression analysis, with measurements of each physical performance (grip strength, 6-m walking time, chair stand time, or one-leg standing test) as objective variables (from Model 1 to Model 4), was performed to estimate the association of physical performance with C7 SVA after adjustment for age, sex, and body mass index. The variance inflation factor was used to investigate multicollinearity in the model. All statistical analyses were performed using JMP version 8 (SAS Institute Inc., Cary, NC, USA). A *p* value of < 0.05 was considered statistically significant.

## Ethical approval

All procedures performed in studies involving human participants were in accordance with the ethical standards of the institutional review board and with the 1964 Helsinki Declaration and its later amendments or comparable ethical standards.

## Consent to participate

Informed consent was obtained from all study participants.

## Data Availability

All data generated or analyzed during this study are available from the corresponding author upon reasonable request.
